# Progress in Surgery

**Published:** 1896-03-14

**Authors:** 


### Progress in Surgery.

### SURGERY OF THE ŒSOPHAGUS AND STOMACH.

**Stricture of the Œsophagus.**—Roberts[1] relates a case in which the contracted portion of the gullet was sufficiently high up for him to perform an œsophagotomy in the neck below the point of constriction, through which the stricture was treated by bougies. Carter[2] also refers to a most difficult case treated by a combined gastrostomy and œsophagotomy, in which the child had been unable to swallow food by the mouth for upwards of five years.

**Foreign Bodies in the Œsophagus.**—Silver's[3] conclusions on the treatment of these cases are: (1) That neglected bodies should be removed at once by operation without making an attempt at extraction through the mouth or pushing them down into the stomach. (2) A foreign body of moderate size should be removed by operation as impaction is considered complete. (3) Where a small sharp body has been swallowed the œsophagus should at once be opened, and where difficult deglutition is present and constitutional symptoms point to septic inflammation. (4) The use of small catheter, bougie, and sponge probang should be condemned. A metal-capped œsophageal sound, with graduated scale, is the only reliable instrument. (5) Where the foreign body is less than thirteen inches from the upper incisors it should be removed by œsophagotomy; if farther down by gastrotomy. (6) Stitching the wound is not essential. The external wound should be left open, although a few sutures can be introduced at the upper corner, leaving it open below. (7) Absolutely no food should be taken by the mouth for the first twenty-four hours, thus giving the planes of the tissues opportunity to become glued together and prevent infiltration and infection of the deep tissues. (8) After the lapse of twenty-four hours liquid food can be given by the mouth, and to prevent its escape from the wound firm pressure can be made with cotton wool over the wound during the act of swallowing. The author gives statistics of 165 cases of œsophagotomy with a mortality of 23 per cent.

**Excision of an Œsophageal Pouch** has been performed by Mixter,[4] there being no constriction in the tube, and the obstruction to the passage of the food being only the thin crescentic edge of the spur between the pouch and the œsophagus, which, pressing against the opposite side of the œsophagus, acted as a valve. Enough of the sac was removed to make the œsophagus a straight tube from the "spur" upwards, the edge being united with catgut stitches. Recovery was perfect.

**Gastrotomy** is so often followed by leakage of the stomach contents and digestion of the surrounding skin that Golding-Bird[5] suggests that the opening into the stomach should be effected rather by dilatation with laminaria tents than by incision, because under such circumstances the orifice always tends to contract. He has tried this method successfully in a case in which the stomach was opened on the fourth day after attachment only enough to admit a No. 10 catheter, and then this small opening was dilated with tents till a short piece of rubber tube, the thickness of the fore-finger, could be introduced and tied *in situ*. The stomach walls contracted round the tube, and from the time of its introduction there was no leakage.

**Disappearing Abdominal Tumours** have from time to time been reported, and Renton[6] records a case where on opening the abdomen he found a large hard irregular tumour extending from the pylorus over the anterior wall of the stomach upwards to the cardiac end. The tumour was fixed in every direction, and had all the appearance of malignancy. The abdomen was, therefore, closed, but the tumour disappeared, and the patient recovered without a bad symptom.

**Hour-glass Contraction of the Stomach** has been treated by Wölfler[7] by gastro-anastomosis. The woman had suffered from stomach symptoms for fourteen years, and an extensive contraction of the middle part of the stomach was found, with distension of both portions of the organ, the constriction being so situated that the first or cardiac half had to lift the contents upwards in order to make them pass through the abnormal opening which lay near the lesser curvature. He made an anastomotic opening between the two halves near the dependent portion of each, and the patient recovered with relief of symptoms. As an aid to diagnosis, Wölfler mentions the possibility of mapping out the distended stomach, and recognising its shape and the occurrence of splashing when nothing can be evacuated from the stomach with the stomach tube, the fluid being in the lower portion.

**The Perforation of a Gastric Ulcer** is, says Barling,[8] marked by extreme pain in the left upper part of the abdomen, as a rule, with collapse. This is speedily followed by rigidity of the abdomen, thoracic respiration, and a subnormal temperature. The course of events then separates the cases into three groups: (1) The acute, (2) the sub-acute, and (3) the chronic. In the acute the outset symptoms are followed by the rapid development of general peritonitis, which, without surgical relief, ends in death in from twelve to forty-eight hours. In the sub-acute, after the shock of perforation has passed off, the symptoms are not marked for some hours or a day or two. Then, owing to further leakage from the stomach, or by the advance of what has for a time been a distinctly localised peritonitis, general septic peritonitis is set up, and without surgical relief death follows in four or five days from the first seizure. In the chronic, after the shock of perforation has passed off, as in the sub-acute cases, there is a period more or less prolonged in which symptoms are slight, and doubt may arise in diagnosis. Then follow the general symptoms of pus formation, and the local signs indicating a gas-containing cavity beneath the diaphragm. Following on these are indications of septic invasion of the thorax, pleurisy, empyema, pneumonia, or lung abscess. After perforation the prospect of recovery without operation is almost hopeless—95 per cent. die, whereas of 37 recorded cases in which an operation was performed 13 recovered. In the treatment of sub-phrenic abscesses, Barling[9] thinks that an opening is required through the wall of the thorax in order to get at the most dependent part of these collections and drain them efficiently. This is best made in the mid-axillary line on the left side, and two or three inches of the eighth rib should be removed. The lung retracts out of the way, and the diaphragm is exposed. Higher up or more posterior than this it is not safe to go. If empyema exists, or lung abscess, these can be now dealt with as seems best. If they are not present the diaphragm should be stitched with a ring of sutures to the edge of the wound in the parietal pleura, and either opened at once, or, perhaps better still, packed and left until the next day, to secure adhesions between the pleural surfaces. On making an opening in the diaphragm, the finger passes into the left sub-phrenic space, just in front of the anterior margin and to the left of the spleen, and a drain should be placed *in situ*. It may be urged that this procedure incurs risk of inducing the very thing we wish to avoid, namely, infection of the thorax. But a study of sub-phrenic abscess shows that the opening from the front is not sufficient for its cure, and that, despite the evacuation by abdominal incision, the pleura or lung nearly always becomes involved. Paul,[10] after sewing up the ulcer in a case of perforation upon which he had operated, was rather afraid that any distension of the stomach might cause the sutures to give way; and he therefore performed a gastrostomy in one stage by placing one of his glass tubes in the stomach and bringing the latter up to the abdominal wall by means of the ligature with which the tube was fastened in the stomach. Selby,[11] in one case, made his incision on the outer side of the left rectus muscle, instead of in the median line. He selected this position under the impression that better access to the whole of the anterior surface of the stomach would be obtained through a wound situated over the middle of that organ than by means of one over its pyloric end. The upper part of the rectus muscle had to be cut across in prolonging the incision, but the whole of the stomach was easily accessible through the wound made. Maurice[12] attaches most importance to the following points: (1) Early operation, which means prompt and accurate diagnosis; (2) the age of the patient and the degree of collapse; (3) the previous history—long standing ulcer means matting of parts and impaired nutrition; (4)

the situation of the ulcer—most perforating ulcers are on the anterior wall, but near the lesser curvature they may be hard to get at; (5) excision seems to be unnecessary, and would be quite impossible in a really difficult case; (6) extensive escape of stomach contents —which means much shock at the time, and probably extensive peritonitis afterwards. Other operative cases are referred to by Barling,[13] Steele,[14] Pollard,[15] Horsley,[16] and Horrocks,[17] but present no features of very special interest.

[1] New York Med. Jour., Nov. 16, 1895. [2] Therapeut. Gazette, May, 1895. [3] New York Med. Record, Oct. 26, 1895. [4] Brit. Med. Jour., Aug. 17, 1895, and Med. News, June 15, 1895. [5] Brit. Med. Jour., Jan. 4, 1896. [6] Ibid., May 25, 1895. [7] Amer. Med. and Surg. Bulletin, Aug. 15, 1895; and Beitr. z. Klin. Chir., 1895, XIII., No. 1, p. 221. [8] Brit. Med. Jour., June 15, 1895, p. 1312. [9] Op. Cit. [10] Lancet, July 6, 1895, p. 31. [11] Lancet, Nov. 30, 1895, p. 1348. [12] Ibidem, Oct. 19, 1895, p. 980. [13] Clin. Jour., Dec 4, 1895, p. 79. [14] Lancet, Aug. 3, 1895, p. 264. [15] Brit. Med. Jour., July 6, 1895, p. 14. [16] Ibidem, July 13, 1895. [17] Lancet, Oct. 2, 1895.

